# Composition of Breast Milk in Women with Obesity

**DOI:** 10.3390/jcm13226947

**Published:** 2024-11-18

**Authors:** Michael G. Ross, Kelly P. Coca, Ana Carolina Lavio Rocha, Bárbara Tideman Sartório Camargo, Luciola Sant’Anna de Castro, Bernardo L. Horta, Mina Desai

**Affiliations:** 1 The Lundquist Institute at Harbor-UCLA, Torrance, CA 90502, USA; 2 Department of Obstetrics and Gynecology, David Geffen School of Medicine, University of California Los Angeles at Harbor-UCLA, Torrance, CA 90502, USA; 3 Paulista School of Nursing, Universidade Federal de São Paulo, São Paulo 04023-062, SP, Brazil; kcoca@unifesp.br; 4 Breastfeeding Center Ana Abrão, Universidade Federal de São Paulo, São Paulo 04037-001, SP, Brazil; aclrocha@unifesp.br (A.C.L.R.); barbara.tideman@unifesp.br (B.T.S.C.); lucastro@unifesp.br (L.S.d.C.); 5 School of Medicine, Universidade Federal de Pelotas (UFPel), Pelotas 96010-610, RS, Brazil; blhorta@ufpel.edu.br

**Keywords:** milk fat, milk calories, milk protein, foremilk, hindmilk, maternal serum lipids, childhood obesity

## Abstract

**Background/Objectives:** Among US breastfeeding women, those with obesity have significantly increased breast milk fat and caloric content from foremilk to hindmilk, with a 4-fold increase in fat content from the first to last milk sample. In view of different dietary norms and nutritional standards, we sought to evaluate the relationship between maternal BMI with breast milk fat and calorie content in women from Brazil, a low–middle-income country. **Methods:** Women who delivered singleton-term neonates were recruited from the Ana Abrao Breastfeeding Center (AABC) and Human Milk Bank at the Federal University of Sao Paulo, Brazil. These women were then studied at 7–8 weeks postpartum. Women were grouped by BMI categories of nonobese (NonOB; BMI 18.5–29.9) and obese (OB; BMI ≥ 30). A breast pump was applied, and milk samples were obtained continuously in 10 mL aliquots from foremilk to hindmilk; samples were analyzed for macronutrients and lipids, and maternal blood was analyzed for serum lipids and glucose. **Results:** As compared to NonOB women, those with OB had significantly higher milk fat in the mid (4.9 ± 0.3 vs. 3.9 ± 0.2) and last hindmilk (6.6 ± 0.4 vs. 5.5 ± 0.3) samples, though not in the first foremilk sample, as compared to NonOB women. In both NonOB and OB subjects, milk caloric and fat content increased 1.5 to 2-fold from foremilk to hindmilk, with the average milk caloric value being 11% greater in OB women. Protein content was significantly increased in all three milk samples (first, middle, and last) in women with OB. **Conclusions**: Although the value of breastfeeding remains clear, these findings may have significant implications for infant nutrition and excessive infant weight gain in women with OB.

## 1. Introduction

Childhood and adult overweight/obesity (OW/OB) prevalence rates are high across all racial groups. There has been a quadrupling in the percentage of children and adolescents with obesity (5.2 to 20%) [1] and severe obesity (1 to 6.1%) over the past five decades, prompting the CDC to extend BMI-for-Age Growth Charts to include four additional percentile curves above the 95th percentile (i.e., BMI can be plotted up to 60 kg/m^2^). Numerous reports have confirmed that childhood obesity is a major risk factor for adult obesity [2,3,4] and the children of overweight/obese (OW/OB) parents have an increased risk of obesity [3], thus creating a generational cycle of obesity.

The cause of the obesity epidemic has often been attributed to a Western diet and reduced work energy expenditure. However, recent animal studies have demonstrated that in utero development may program offspring predisposition to weight gain and obesity, in part via enhanced appetite neural pathways and adipogenesis [5,6,7]. Our studies [5,6] and others [8,9] have demonstrated that development in a maternally obese environment exposing the fetus to increased fatty acids and oxidative stress may shift hypothalamic arcuate neural progenitor cells toward a predominance of appetite (neuropeptide Y) as opposed to satiety (pro-opiomelanocortin) neurons, with a consequence of newborn and adult hyperphagia. In a parallel process, early induction of adipogenic transcription factor (peroxisome proliferator-activated receptor gamma; PPARγ) results in enhanced adipogenesis with increased propensity for lipid storage and hypertrophic adipocytes [7,10]. During the newborn period, a potential remodeling of arcuate appetite neurons [11] may moderate in utero programming responses. However, continued exposure to an obese-associated environment may potentiate the programming effects. The evidence of lactational programming is clearly demonstrated in rodents. Mice born to and nursed by obese dams demonstrate early life and adult obesity [12,13]. However, offspring of obese dams who are cross-fostered and nursed by control dams grow to normal weight as adults [12], whereas offspring of control dams who are nursed by obese dams develop adult obesity [12].

In humans, the importance of “lactational programming” [14] is evidenced by the finding that rapid weight gain between birth and 4 months is associated with a 5-fold risk of obesity at age twenty [15].

Infant nutrition, whether by breast milk or formula, has important consequences for later life health. The American Academy of Pediatrics and the World Health Organization (WHO) recognize exclusive human milk feeding as the normative standards for infant feeding for the first six months after birth and recommend continued breastfeeding until two years of age and beyond [16]. Nearly 80% of women initiate breastfeeding at birth, and 48–60% have some breastfeeding continuing at 6 months [17,18,19]. Although the enhanced nutritional value of breast milk vs. formula is well accepted, controversy remains as to whether breastfeeding reduces the incidence of childhood obesity [20,21,22]. Maternal behavioral feeding patterns in response to infant fussiness, bottle-feeding of human breast milk, and social determinants may contribute significantly to infant weight gain. A critical, though often unaddressed, factor contributing to excessive infant weight gain may be the caloric and fat composition of human milk.

In a recent study, we demonstrated that among US breastfeeding women, those with obesity had increased breast milk fat and caloric content. The results suggested that both maternal obesity and diet-induced increases in serum triglycerides impact the higher milk fat content. In comparison to American dietary guidelines, Brazilian nutritional guidelines advise people to choose fresh foods, prefer healthier types of fat, limit trans-fat intake, and minimize processed foods [23]. In view of the potential dietary differences, we sought to determine if the association of maternal obesity with breast milk fat content was present among Brazilian women.

## 2. Materials and Methods

### 2.1. Study Participants

**Figure 1:**
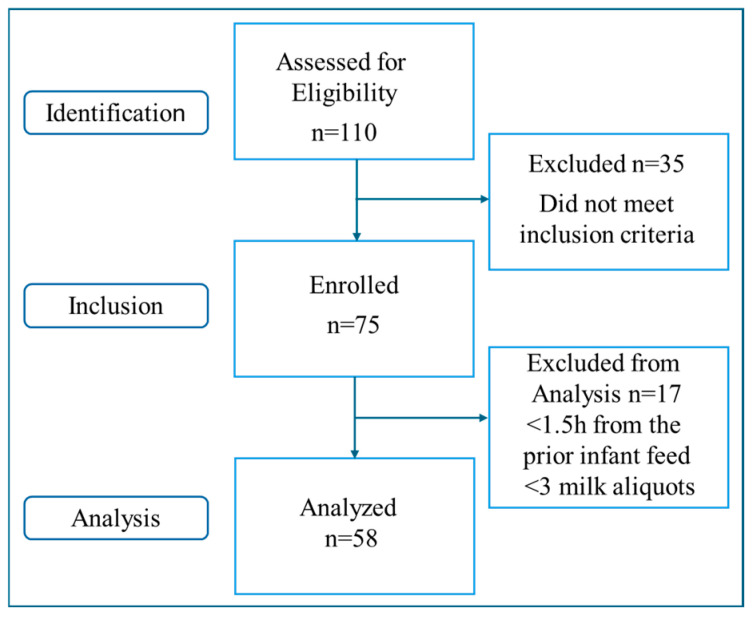
STROBE flow chart of study participants.

This study was approved by the Research Ethics Committee at Universidade Federal de São Paulo (Study protocol # 4.454.132, approval date 10 December 2020). Women delivering singleton-term pregnancies were recruited at Ana Abrao Breastfeeding Center (AABC) and Human Milk Bank, Federal University of Sao Paulo, Brazil. Following informed consent, staff interviewed all subjects and obtained demographic information (maternal age, ethnicity, pre-pregnancy height, and weight) while the electronic medical records were referenced for additional medical data (weight at the last prenatal visit, gestational age at delivery, pregnancy complications (including gestational diabetes), medications, mode of delivery, and baby’s length and weight at birth and discharge and gender). Exclusion criteria include women with pregestational diabetes, prior breast surgery, breast implants, flat/inverted nipples that inhibit breastfeeding, low birth weight infants (<2500 g), or infant tongue-tie. Women were selected for BMI (based on pre-pregnancy reported weight): 18–29.9 (Nonobese, NonOB) and ≥30 (Obese, OB). All subjects were planning on exclusive breastfeeding for at least two months. Studies were performed at 7–8 weeks postpartum (mature milk). Among the 110 women screened, 75 met the inclusion criteria, and 58 qualified for analysis (Figure 1).

All studies were performed at Ana Abrao Breastfeeding Center between 10 am and 12 pm to maintain consistent timing of breast milk samples and avoid circadian effects. Mothers refrained from eating or drinking for >1 h prior to the study and had refrained from breastfeeding for at least 1.5 h from the prior infant breastfeeding, which was from only one breast. Maternal blood samples were drawn for analysis prior to placement of the breast pump.

The breast opposite that was used for the prior feed (preceding the study) was used for breast milk sampling. The areola and nipple were cleansed, and an electrical pump (Medela, McHenry, IL, USA) was applied to the breast. Pumped milk samples were obtained continuously in 10 mL aliquots, and the process continued until the primary breast was emptied or there was no further milk production. Milk samples were analyzed using the Miris (Uppsala, Sweden) milk analyzer.

### 2.2. Blood Analysis

Serum samples were sent to the Biochemical and Clinical Pathology Laboratory, Sao Paulo, for analysis of lipid panel (triglycerides, total cholesterol, LDL-cholesterol, HDL-cholesterol, non-HDL cholesterol, cholesterol/HDL ratio) and glucose.

### 2.3. Milk Analysis

Milk fat, protein, and carbohydrate content were assessed using the ‘Miris Human Milk Analyzer.’ All samples were kept at 40 °C (Miris HMA, Sweden ), homogenized for 1.5 s/1 mL (Miris Ultrasonic Processor), and samples injected into the flow cell and measured in triplicate. For the determination of fat, protein, and carbohydrate content, the Miris system utilizes semi-solid mid-infrared (MIR) transmission spectroscopy for measurement of functional carbonyl groups (5.7 µm), amide groups (6.5 µm), and hydroxyl groups (9.6 µm), respectively. True protein is adjusted for non-protein nitrogen (crude protein multiplied by 0.8), and total carbohydrate content includes both lactose and human milk oligosaccharides. Total solids are measured in a drying oven, and the analyzer provides a calculation of energy (kcal per 100 mL) using conversion factors of 4.0 (carbohydrate), 9.25 (fat), and 4.4 (protein).

### 2.4. Statistical Analysis

An unpaired *t*-test was used for maternal blood and characteristic data analysis. For the breast milk composition from each woman, the first, middle, and last milk samples were analyzed by repeated measures of ANOVA with post hoc analysis to assess differences between the two groups. Linear regression was used to test if maternal milk volume was correlated with milk fat content. Normality was tested with the Shapiro–Wilk test. Values are expressed as means ± SEM (standard error mean). All analyses included n = 40 nonobese (BMI, 18.9–29.9) and n = 18 obese (BMI, ≥30) mother–infant dyad.

## 3. Results

### 3.1. Maternal Characteristics

Women were enrolled at the Ana Abrao Breastfeeding Center (AABC) and Human Milk Bank, Federal University of Sao Paulo, Brazil, and were not provided any specific hydration guidelines or supplements/medication. A total of 40 NonOB (BMI 24.9 ± 0.5) and 18 OB (BMI 35.2 ± 1.1) women were studied (Table 1). There were no significant differences in maternal fasting (1 h) serum value between NonOB and OB women (Table 2). The infant gender was 24 male and 34 female. As there were no sex differences, the combined data are presented in Table 3. The infants of women with OB had significantly higher birth weights than infants of NonOB (Table 3).

### 3.2. Milk Composition

**Figure 2:**
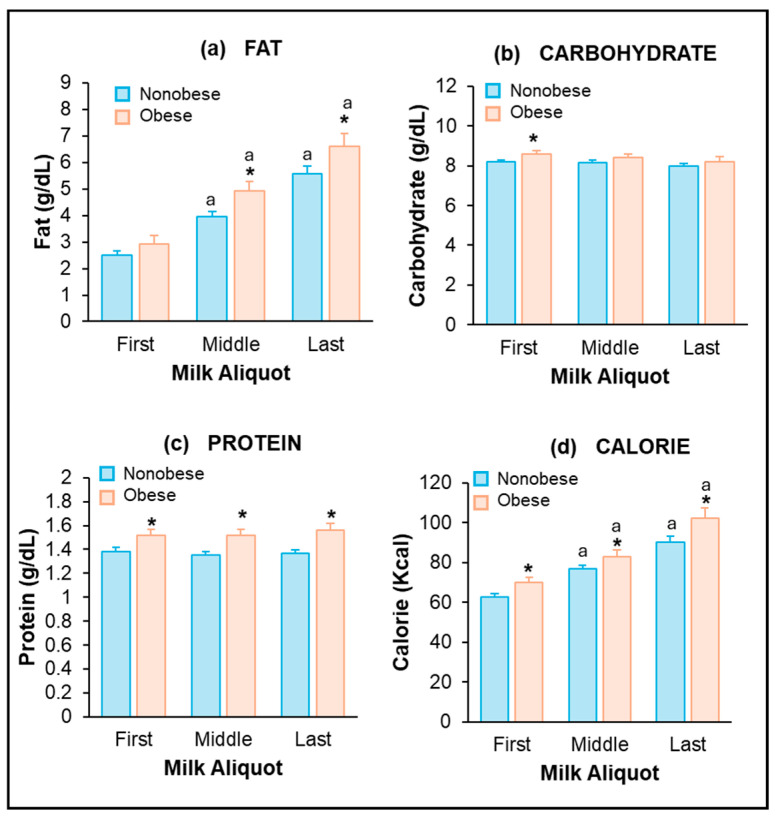
Maternal BMI and milk composition: (**a**) Fat, (**b**) carbohydrate, (**c**) protein, (**d**) calorie content of first, middle, and last milk samples from NonOB and OB women. Values are mean ± SEM of n = 40 (NonOB) and n = 18 (OB) groups. * OB vs. NonOB; ^a^ middle, last vs. first milk sample.

The breast volume (i.e., total pumped milk volume) was similar in the NonOB and OB groups. Milk composition was examined in the first, middle, and last 10 mL samples from the pumped breast. Women with OB had significantly higher fat content in the middle and last, but not first, milk samples as compared to NonOB women (Figure 2a; F(1, 2) = 6.5, *p* = 0.014). Conversely, carbohydrate content was significantly higher only in the first milk sample of women with OB (Figure 2b, F(1, 2) = 3.66, *p* = 0.053). Protein and calorie content were significantly increased in all three milk samples (first, middle, and last) in women with OB (Figure 2c,d; protein: F(1, 2) = 9.9, *p* = 0.003; calorie: F(1, 2) = 4.6, *p* = 0.030). Caloric content values were 11% greater in women with OB when averaged over the three milk samples.

In both NonOB and OB women, from the first to last sample, milk caloric and fat content increased 1.5 to 2-fold (Figure 2a,d), with mid-samples representing intermediate values. Carbohydrate and true protein content remained unchanged from the first to last sample (Figure 2b,c).

### 3.3. Maternal Serum, Milk Composition, and Milk Volume

Linear regression showed no correlation between plasma cholesterol, triglycerides, and glucose with foremilk and hindmilk content.

**Figure 3:**
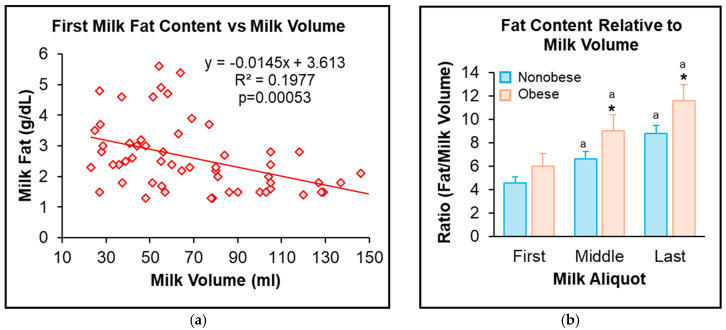
Milk volume vs. first milk fat content: (**a**) Linear regression of n = 58 of combined NonOB and OB groups. (**b**) First, middle, and last milk fat content was adjusted relative to milk volume in women with NonOB and OB. Values are mean ± SEM of n = 40 (NonOB) and n = 18 (OB) groups. * OB vs. NonOB; ^a^ middle, last vs. first milk sample.

Linear regression analysis of milk volume versus macronutrients was performed to assess the effects of volume contributing to milk composition. Notably, first milk fat and calorie content (Figure 3a) were negatively correlated with milk volume [fat: R^2^ = 0.145, F(1, 56), *p* = 0.000; calorie: R^2^ = 0.141, F(1, 56), *p* = 0.000]. Consistent with the above results, women with OB had significantly higher milk fat and calorie content even when expressed relative to milk volume (Figure 3b). Conversely, milk protein (R^2^ = 0.0276, *p* = 0.212) and carbohydrate (R^2^ = 0.002, *p* = 0.913) showed no correlation with milk volume.

## 4. Discussion

The present results confirm that women with OB have higher breast milk fat and caloric content than NonOB women. Among all women, milk fat content increased 1.5 to 2-fold from foremilk to hindmilk, in parallel to an increase in caloric content. In comparison to Brazilian women, we previously reported that US women demonstrated a lower concentration of milk fat in the first foremilk sample (1.8 vs. 2.6) and a slightly higher concentration in the last hindmilk sample (6.3 vs. 5.9), resulting in a 3–4 fold [24] increase in milk fat content from foremilk to hindmilk in US women. Further differences among the populations are evident as Brazilian, though not US, women with OB demonstrated increased milk protein content, from foremilk to hindmilk.

Although the milk metabolome, proteome, lipidome, and small bioactives contribute to infant well-being, macronutrients are the primary determinants of milk caloric intake and infant weight gain. Human milk is a complex biological fluid of more than 200 identified components, including solutions, colloids, membranes, globules, and live cells [25]. On average, milk contains 0.8–0.9% protein, 3–5% fat, 6.9–7.2% carbohydrates, and 0.2% minerals and averages 66 kcal/100 mL [26,27,28,29] with an interquartile range of 62.0 to 72.5 kcal/100 mL, reflecting the significant individual variance. Milk carbohydrates are primarily lactose, as well as an array of oligosaccharides. Milk fat represents the variable component of caloric content, contributing 40–50% of human milk calories [30], with triglycerides accounting for 98% of milk lipid content. Fatty acid composition is highly variable, with both maternal BMI and diet being important determinants of milk polyunsaturated fatty acids, lipid content, and total caloric content [28,29,31,32,33,34,35,36]. In mature human milk, the majority of triglycerides consist of long-chain fatty acids (oleic, 20–35%; palmitic, 18–23%; linoleic, 8–18%) [37] and are dependent in part on maternal diet and genetics [33].

The sources of milk triglycerides include endogenous fat stores, dietary lipids, and de novo fatty acid synthesis from mammary cells. Breast milk triglycerides result from two sources: de novo mammary epithelial cell synthesis and endogenous mammary epithelial cell fatty acid uptake from serum. De novo mammary epithelial cell synthesis contributes primarily to short-chain and medium-chain fatty acids (C6–C12), with limited production of C14–C16 fatty acids [30,37]. Acetate and β-OH butyrate, the primary carbon sources, are absorbed through the mammary epithelial cell basolateral membrane [38], and synthesis is primarily catalyzed by fatty acid synthase (FAS) and acetyl CoA carboxylase (ACACA) [39]. Among the genes and transcription factors that regulate milk fat biosynthesis are the family of sterol regulatory element-binding proteins (SREBP) and the peroxisome proliferator-activated receptor γ (PPAR) [40]. As mammary epithelial cells have a limited ability to synthesize C18 fatty acids, endogenous fat stores and diet contribute to long-chain fatty acids. These long-chain fatty acids, which are primarily carried by chylomicrons (intestinal absorption) or hepatic very low-density lipoproteins, are transported to mammary epithelial cells, where apolipoprotein B interacts with mammary epithelial cell surface lipoprotein receptors. Following hydrolysis by mammary epithelial cell lipoprotein lipase (LPL), which increases dramatically with lactation [41,42], fatty acids enter the cell and can be desaturated (stearoyl-CoA desaturase) and/or converted into TGs, phospholipids, or cholesterol esters. Thus, the uptake of TG fatty acids is dependent upon mammary LPL as well as pathways of fatty acid transport, trafficking, production, and secretion in milk [43]. In our prior study of US women, breast milk of obese subjects demonstrated increased levels of both short-chain and long-chain fatty acids [24], representing both of the major pathways for milk fat content. Thus, the increased milk fat content is likely a complex mix of dietary, endocrine, and metabolic consequences of obesity. Short-chain fatty acids comprise <1% of total fatty acids but serve to induce newborn satiety and as critical precursors. Medium-chain fatty acids comprise ~12% of total fatty acids and are preferentially absorbed and metabolized by neonates. In mature human milk, the majority of TGs in the milk fat globule core consist of long-chain fatty acids. Among the long-chain fatty acids are diet-dependent essential fatty acids.

Hormones also play a significant role in TG synthesis. Studies confirm a significant role of insulin in milk de novo fatty acid synthesis as well as the incorporation of preformed fatty acids into milk TGs [44,45]. Evidence suggests that insulin resistance occurs unequally among primary insulin-target sites, while the mammary breast is extremely sensitive to insulin during lactation [46], with its receptor increasing by 2.5-fold [47]. Accordingly, mammary gland insulin receptor knockout downregulates an array of genes involved in mice milk lipid synthesis, milk fat globule formation, and milk lactose synthesis, with reduced lipid droplets and casein staining [48]. In vitro insulin treatment of mouse mammary epithelial cells induces gene expression of transcription factor SREBP1, phosphorylation of Akt [47], and lipid uptake (LPL) and synthesis (ACACA, FAS) enzymes.

Remarkably, there is little understanding of the mechanism for the marked increase in milk fat from foremilk to hindmilk. Mizuno et al. [49] demonstrated that the mean fat globule size was not different between hindmilk and foremilk (despite a doubling of the fat content), indicating that the increased fat content was a consequence of a greater number of milk fat globules. However, in daily cows, milk fat globule size increased in relation to milk fat in one study [50] but not in another study [51]. Thus, the actual contribution of milk fat globule size or number to milk fat contents remains unclear.

Based on the present results, maternal obesity is a major factor contributing to increased breast milk fat content. Our recent study suggested that maternal serum triglyceride concentration may also impact foremilk breast milk fat content [24]. The absence of significantly increased serum triglyceride levels among OB Brazilian subjects correlates with similar levels of foremilk fat content among OB and NonOB women, whereas OB Brazilian mid and hindmilk fat levels were increased. These results suggest that serum triglycerides may have a greater influence on foremilk than on hindmilk fat content. Maternal diet and, thus, plasma triglyceride composition effects on breast milk fat content occur over a time period of at least several hours [52]. As we assessed only plasma triglycerides at the time of the morning pumping, it is unknown if concentration differences in the hours prior to pumping contributed to the ultimate milk content. The higher foremilk and lower hindmilk fat content as compared to US women may be a reflection of population dietary differences.

Similarly, the increased milk protein content in Brazilian OB women, though stable from foremilk to hindmilk, may be a consequence of diet, as maternal BMI was positively associated with a higher protein level in human milk as well as with a higher dietary protein intake [53]. Milk proteins consist of whey (α-lactalbumin, lactoferrin, β-lactoglobulin, albumin, and immunoglobulins) and casein (phosphoproteins) in addition to select hormones (leptin, ghrelin). Milk protein synthesis is affected by the amino acid supply and profile with essential amino acids most important in promoting milk protein synthesis in dairy cows [54]. Increasing the supply of essential amino acids may enhance cow milk protein content [53,55], though maternal protein intake does not appear to impact human milk protein [56]. However, Binder et al. [53] demonstrated that maternal BMI was positively associated with higher human milk protein levels as well as with a higher dietary protein intake. It is possible that the amino acid intake, rather than simply total protein intake, impacts milk protein content. In future studies, we plan to assess maternal dietary intake over the 24–48 h prior to each milk assessment.

These findings may have significant implications for infant nutrition, weight gain, and perhaps childhood obesity. Breastfeeding patterns may vary from paired feeding (feeding from each breast at each feed) to alternate breastfeeding (one breast primarily at each feed). With paired feeding, the infant may preferentially receive lower fat foremilk. Whether the lower caloric intake would be compensated by an infant appetite-driven increase in milk volume intake is unknown. Similarly, it is unknown whether subsequent nursing after paired feeding again results in the provision of lower-fat foremilk or whether the higher-fat “hindmilk” is released first, effectively becoming foremilk. Whereas it is possible that infants of women with OB reduce the intake of the higher milk fat and caloric content, even a small mismatch (<0.5%) in intake vs. expenditure will lead to weight gain over time [57]. The finding that child weight-for-age Z score is increased in women with increased breast milk fat [58] suggests a linkage between maternal OB, increased breast milk fat content, and excessive infant weight gain. As there are few comprehensive studies of breast milk composition (foremilk to hindmilk) and very limited and relatively imprecise studies of the volume of milk ingested, we have scant knowledge of the nutritional intake of exclusively breastfed infants.

OB women had a slightly, but not significantly, decreased volume of breast milk obtained by pumping. There have been numerous reports of challenges of breastfeeding among OB women, a result of body habitus and breast size as well as infant positioning and latching [59]. Although only a single sample, these results suggest that perceived difficulties in OB breastfeeding may not be a consequence of reduced milk volume.

Animal studies confirm the importance of lactational programming. Obese/high-fat diet mice produce high-fat milk, which contributes to early-life obesity [60,61,62]. Together with the present data indicating an 11% increase in milk caloric density of women with OB, these results raise a concern about the potential impact of high-fat/high-calorie milk on early life weight gain. Thus, lactation is not only a critical period for the infant’s development and programming effects; it also provides a window of opportunity to correct functional deficits that may have occurred from prenatal challenges.

## 5. Conclusions

The present study utilized a method of pumping a full breast (not the breast used for the immediate prior infant feed) with aliquots of milk sampled every 10 mL. Nearly all published studies of breast milk composition have used samples of a single-timed specimen during feeding, an aliquot taken from the entire milk volume, or a random timed sample. In view of the 2-fold increase in breast milk fat content and the increase in protein content from foremilk to hindmilk, it is important for future studies to standardize milk collection protocols as well as details as to the time period from a prior feed.

We remain committed to the value of exclusive breastfeeding. However, it is important to recognize the variance of breast milk among individuals, whether impacted by maternal BMI, diet, protein, carbohydrate, or fat metabolism. Beyond fat and caloric content, the more than 200 identified components [37] include essential fatty acids (e.g., DHA), an array of human milk oligosaccharides, critical proteins, immunoglobulins, and small bioactives. A better understanding of both the nutrient composition and the infant intake can potentially optimize nutrition during this critical portion of infant life.

## Figures and Tables

**Table 1:** Maternal Characteristics.

	Nonobese (18.9–29.9) n = 40	Obese (≥30) n = 18
Age (years) Prepregnancy BMI (kg/m^2^)	24 ± 1 24.9 ± 0.5	27 ± 2 35.2 ± 1.1 *
Vaginal delivery (n)	32	9
Cesarean section (n)	8	9
Medication (n)	12	8
Gestational diabetes (n)	5	4
Gestational weight gain (kg)	12.3 ± 0.8	5.6 ± 1.6

Values are mean ± SEM; * *p* = 0.000.

**Table 2:** Maternal Serum Values and Milk Volume.

	Nonobese (18.9–29.9) n = 40	Obese (≥30) n = 18
Glucose (mg/dL)	72.2 ± 1.1	73.1 ± 2.7
Triglycerides (mg/dL)	118.5 ± 9.7	126.7 ± 11.7
Total cholesterol (mg/dL)	205.1 ± 8.8	216.7 ± 16.1
LDL-cholesterol (mg/dL)	125.6 ± 6.9	127.6 ± 13.5
HDL-cholesterol (mg/dL)	55.8 ± 2.4	63.8 ± 3.9
Non-HDL cholesterol (mg/dL)	149.3 ± 7.8	152.9 ± 13.4
Cholesterol/HDL Ratio	23.6 ± 1.9	25.3 ± 2.3
Total milk volume (mL)	75.2 ± 6.1	69.5 ± 8.4

Values are mean ± SEM.

**Table 3:** Infant Characteristics.

	Nonobese (18.9–29.9) n = 40	Obese (≥30) n = 18
Male	17	7
Female	23	11
^1^ Birth Weight (g)	3266 ± 52	3448 ± 86 *

^1^ Birth weight is combined as there are no significant differences between sexes. Values are mean ± SEM; * *p* = 0.034.

## Data Availability

The data presented in this study are available from the corresponding author upon reasonable request. The data are not publicly available due to privacy or ethical restrictions.
